# Delivering CRISPR to the HIV-1 reservoirs

**DOI:** 10.3389/fmicb.2024.1393974

**Published:** 2024-05-15

**Authors:** Theodore E. Gurrola, Samuel N. Effah, Ilker K. Sariyer, Will Dampier, Michael R. Nonnemacher, Brian Wigdahl

**Affiliations:** ^1^Department of Microbiology and Immunology, Drexel University College of Medicine, Philadelphia, PA, United States; ^2^Center for Molecular Virology and Gene Therapy, Institute for Molecular Medicine and Infectious Disease, Drexel University College of Medicine, Philadelphia, PA, United States; ^3^Department of Microbiology, Immunology, and Inflammation and Center for Neurovirology and Gene Editing, Temple University Lewis Katz School of Medicine, Philadelphia, PA, United States; ^4^Sidney Kimmel Cancer Center, Thomas Jefferson University, Philadelphia, PA, United States

**Keywords:** HIV, latent viral reservoir, CRISPR, vector design, biodistribution

## Abstract

Human immunodeficiency virus type 1 (HIV-1) infection is well known as one of the most complex and difficult viral infections to cure. The difficulty in developing curative strategies arises in large part from the development of latent viral reservoirs (LVRs) within anatomical and cellular compartments of a host. The clustered regularly interspaced short palindromic repeats/ CRISPR-associated protein 9 (CRISPR/Cas9) system shows remarkable potential for the inactivation and/or elimination of integrated proviral DNA within host cells, however, delivery of the CRISPR/Cas9 system to infected cells is still a challenge. In this review, the main factors impacting delivery, the challenges for delivery to each of the LVRs, and the current successes for delivery to each reservoir will be discussed.

## 1 Introduction

Due to the inherent inability for the current treatment and cure strategies to overcome the challenges that HIV-1 disease presents, there is a need for new strategies to achieve a cure of this therapeutically controlled chronic disease. This is complicated by a number of associated comorbid conditions including aging, cancer, infection by other secondary pathogens, and, substance abuse. The newest of these therapeutic strategies is the use of cell and gene therapy. The main gene editing technologies currently in use include transcription activator–like effector nucleases (TALENs), zinc finger nucleases (ZFNs), and the clustered regularly interspaced short palindromic repeats (CRISPR) system with the latter of these having been shown to be the most effective approach (Khalili et al., 2017; Atkins et al., 2021).

The CRISPR system is composed of a Cas nuclease associated with a small RNA molecule and was originally discovered as a key component of a bacterial defense system directed against bacteriophages. Since its discovery in 1987 (Ishino et al., 1987), CRISPR has been redesigned for a number of different gene-editing applications (Cho et al., 2013; Cong et al., 2013). While ZFNs and TALENs operate by binding a protein to the desired DNA target, the CRISPR system operates by creating a complementary base pair between the CRISPR’s RNA and the desired DNA sequence.

**Figure 1:**
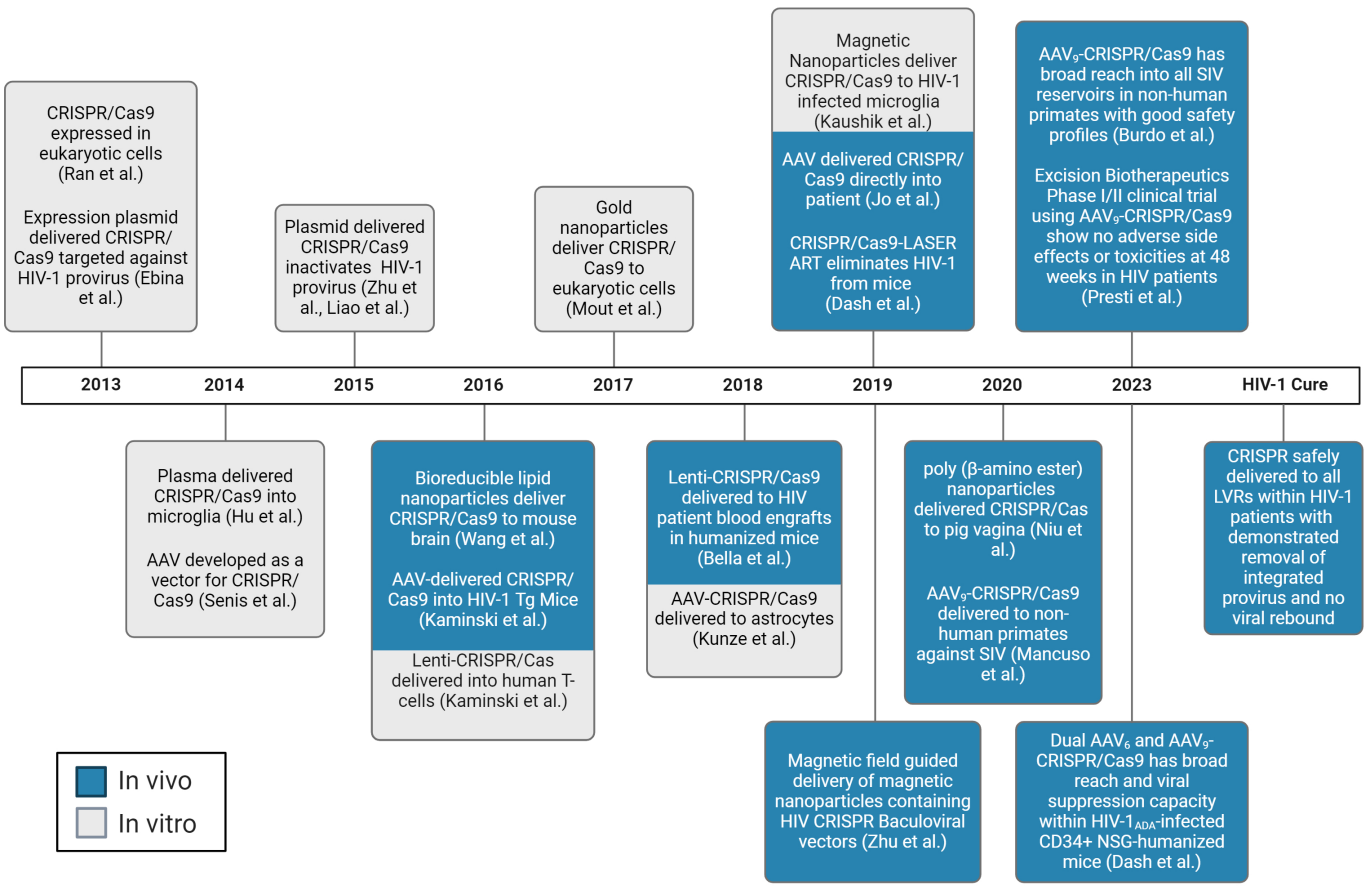
Chronological progress of CRISPR/Cas delivery to LVR. Experiments demonstrated to deliver the CRISPR/Cas system to living subjects are depicted in blue. Experiments demonstrated to deliver the CRISPR/Cas system to *in vitro* are depicted in gray. AAV, adeno-associated virus. Created with BioRender.com.

In the CRISPR technology, a guide RNA (gRNA) directs the system to a specific DNA sequence through a 20-nucleotide region that is complementary to the genomic DNA target, known as the protospacer. Partial mispairing is tolerated in the PAM distal region of the gRNA (Hsu et al., 2013; Doench et al., 2016). It has been thought this may increase the likelihood of off-target cleavage. Indeed, distinct targets experience significantly varied amounts of off-target effects, possibly due to varying gRNA design (Dampier et al., 2018; Atkins et al., 2021). However, for HIV, this effect has been shown to allow for gRNA design that may increase the spectrum of quasispecies targetable in and between people living with HIV (PWH) (Dampier et al., 2017, 2018, 2024; Sullivan et al., 2019; Chung et al., 2020, 2021; Allen et al., 2023). The *Streptococcus pyogenes* (Sp)Cas9 nuclease is the most widely employed of the CRISPR systems, although several other Cas9 strains and Cas molecules are being explored depending on the desired outcome. The popularity of the CRISPR system has been growing exponentially in recent years since the discovery of its gene editing capabilities. Indeed, while it was initially discovered in 1987, the progression toward its use as a therapeutic agent has occurred as a rapid pace. However, while the CRISPR/Cas system is a powerful tool to edit genetic information, it is only as strong as the weakest link in the process of treatment administration. While Cas9 itself is effective when it is present in the cell of interest, delivering the gene editing tool throughout a host’s body to every cell of interest has been extremely challenging. The factors affecting delivery of expression vectors is incredibly complex, but there has been significant progress in optimizing transport of a vector’s payload, including that of CRISPR. A timeline of the seminal achievements in CRISPR delivery is shown in Figure 1.

In this review, we focus on recent progress in CRISPR-based therapeutic approaches directed against HIV-1 as an improvement on current therapeutic strategies incapable of eradicating HIV-1 from its integrated format. The CRISPR/Cas system is well known for its effective DNA-editing capacity, and ease of use, making it the system of choice in many research applications including targeting the HIV-1 proviral genome or editing chemokine receptor type 5 (CCR5) out of cells. However, one of the main challenges with the use of CRISPR in a clinical setting is the delivery of the system to the anatomical and cellular reservoirs of interest. This review will discuss efforts to deliver the CRISPR system to these anatomical and cellular reservoirs.

## 2 Factors affecting expression vector delivery

The journey of vectors carrying HIV-1 treatments to the LVR is fraught with many physical and biological barriers. The journey typically begins after intravenous (IV) injection when the vector is almost immediately met with plasma proteins and the mononuclear phagocyte system (MPS) (Gustafson et al., 2015). Following this clearance system, the second main set of clearance systems are the liver, spleen, and kidneys, which clear almost all vectors introduced to the body (Casals, 2008). Furthermore, while in circulation, vectors experience different degrees of shear stress, due to the various diameters of the vasculatures, which can strip vectors of their surface coating, and damage a vector or its cargo (Hosta-Rigau and Stadler, 2013; Jarvis et al., 2018), a concern of particular interest due to the sensitive and multicomponent nature of the CRISPR/Cas system. This problem is augmented by the prevalence of hypertension in people living with HIV-1 (Xu et al., 2017). Shear stress can also preclude extravasation by preventing any vector from marginating and adhering to vascular walls in order to extravasate toward a target tissue (Hosta-Rigau and Stadler, 2013; Blanco et al., 2015; Cooley et al., 2018; Jarvis et al., 2018; Khor et al., 2018).

A vector able to successfully overcome these obstacles then must marginate from circulation, adhere to the vascular walls, and internalize within the desired tissue and/or cell type. Of course, this is no small task and is currently complicated by too many factors to fully elucidate the process. Physiologically based pharmacokinetic (PBPK) modeling has attempted to shed much needed light on the process of delivery to tissues of interest, but even the latest models cannot consistently account for the enormous variations between tissues, microenvironments, and individuals (Yuan et al., 2019). Indeed, the barriers faced by a vector generally further complicate delivery in response to disease states, such as inflammation and the multiple stages of HIV-1 infection (Lawrence et al., 1999).

### 2.1 Effect of vector size on biodistribution

The main factor determining size selectivity in each tissue type is the type of blood capillary wall present, which is generally composed of three layers in most types of tissue blood capillaries. These layers are the endothelial glycocalyx layer (EGL) facing the lumen of the blood vessel (Weinbaum et al., 1992; Squire et al., 2001), a layer of endothelial cells is below this (Kanwar and Farquhar, 1979; Charonis and Wissig, 1983), and the bottom layer is the basement membrane layer facing the interior of the tissue (Rhodin, 1962; Elfvin, 1965; Luft, 1966; Friederici, 1968; Brightman and Reese, 1969; Maul, 1971; Yee and Revel, 1975; Mason et al., 1979; Bearer and Orci, 1985). Of course, particles must pass through all layers in order to successfully extravasate from the blood into tissue. The most restrictive layer of the capillary wall determines the upper limit of particle size allowed to extravasate (Sarin, 2010). Exceptions exist to this general principle due to disease states that cause endothelial dysfunction, leading to increased permeability of the blood capillary wall, such as acute inflammation (Daiber et al., 2017), chronic inflammation (Castellon and Bogdanova, 2016), and HIV-1 infection (Mazzuca et al., 2016). Random openings and heterogeneous cell layers can also permeabilize the blood capillary wall to particles sizes not generally allowed into a given tissue type. These exceptions may explain the observations of vectors with larger than permitted sizes into more restrictive tissue types, which is discussed in more detail in the LVR section.

**Figure 2:**
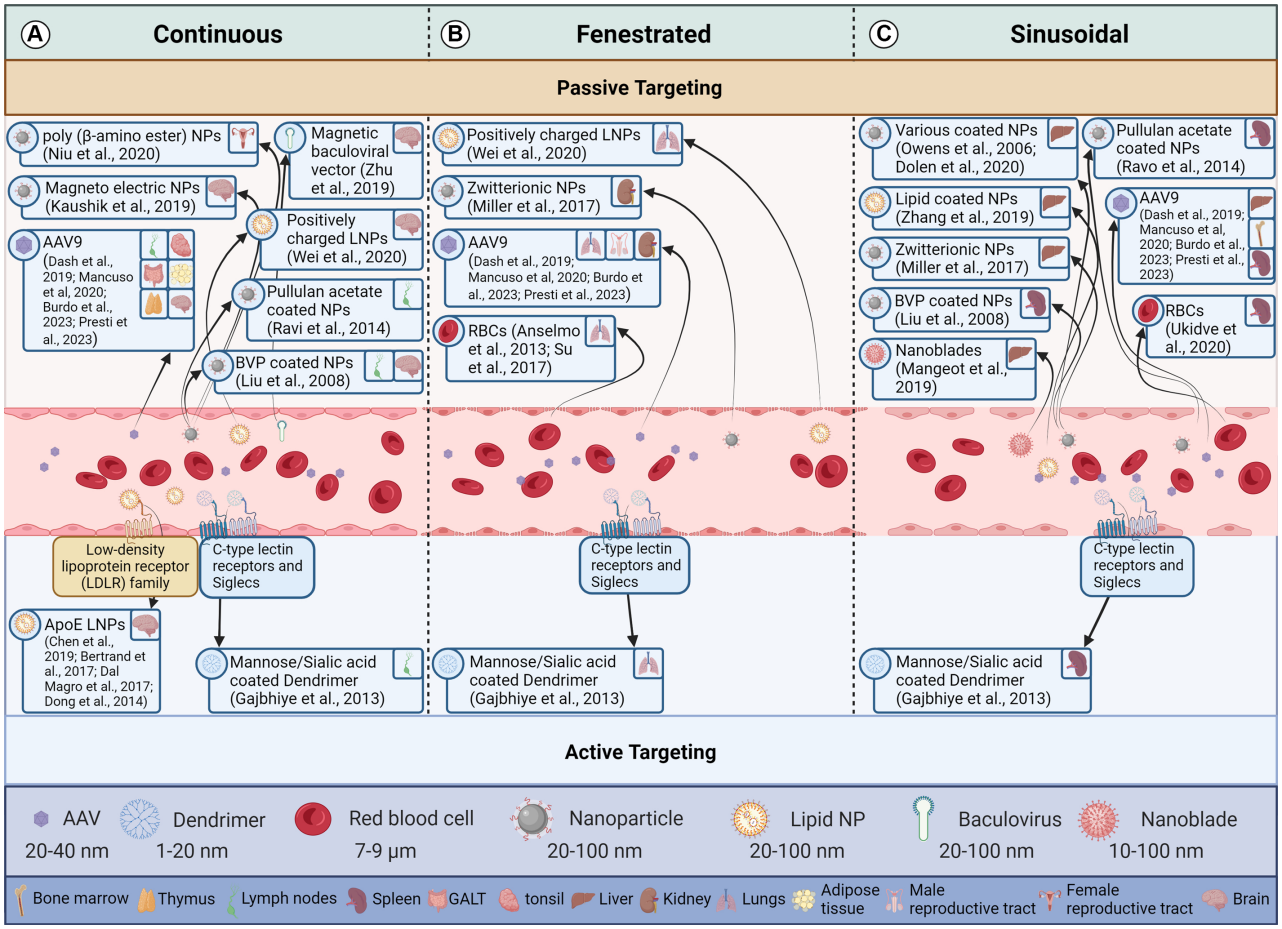
Effect of endothelial cell type and vector attributes on a vector reaching the target tissue. This figure illustrates how the structure of endothelial cells lining blood vessels influences the delivery of therapeutic vectors to target cells through both passive and active transport mechanisms. The three panels represent different types of blood capillaries. Arrows indicate the movement of a vector from the blood stream to a particular latent viral reservoir (LVR). Vectors demonstrated to deliver to a LVR tissue are in the same box for all blood capillary types. **(A)** Continuous Endothelium: These capillaries have a continuous, tightly sealed cell layer with minimal gaps. Passive diffusion of small molecules (<5 nm) and lipophilic vectors is possible, but larger molecules and hydrophilic vectors struggle to penetrate. Active transcytosis, where the vector is shuttled across the cell by the endothelium, can occur but is limited. **(B)** Fenestrated Endothelium: These capillaries have numerous small pores (fenestrae) in the endothelial layer, allowing for passive diffusion of macromolecules (up to 70 nm) and some vectors. However, larger vectors and those lacking specific targeting moieties may still struggle to reach target cells. Active transcytosis is more efficient in fenestrated capillaries due to the increased surface area and porous nature. **(C)** Sinusoidal Endothelium: These capillaries have the largest gaps between endothelial cells and lack a continuous basement membrane. This allows for passive diffusion of even large molecules and vectors, making them ideal for delivery to cells like liver cells. However, non-specific binding and extravasation to unintended tissues can be a challenge. Active transcytosis is also possible in sinusoidal capillaries but may be less critical due to the ease of passive diffusion. Created with BioRender.com. References for the relevant papers are included in the box’s for each vector delivery illustration.

The three main capillary types are continuous, fenestrated, and sinusoidal or discontinuous (Figure 2). Each type has a different permeability to the crossing of solutes from blood, with continuous capillaries being almost impenetrable to vectors under normal conditions and sinusoidal being the most permeable (Aird, 2007a,b). The diameter of pores differs between tissue types. The exact type of capillary present in each reservoir is discussed in the respective tissue sections.

### 2.2 Effect of vector charge on biodistribution

**Figure 3:**
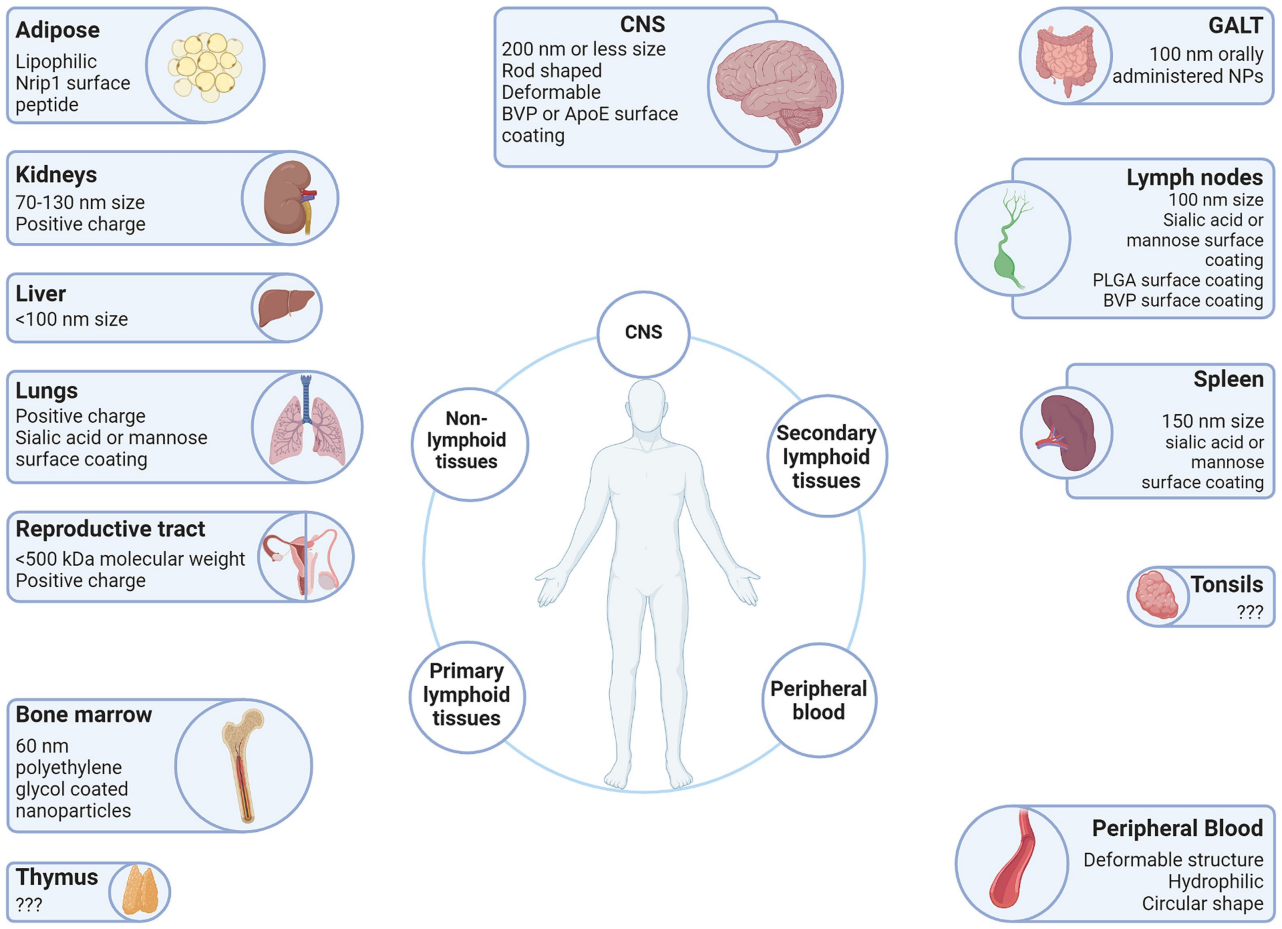
Vector attributes that improve delivery to the LVR. Anatomical sites associated with the HIV-1 LVR are depicted with beneficial qualities of vectors that have been demonstrated to facilitate entry into each anatomical LVR. Created with BioRender.com.

Vector surface charge is quantified in millivolts (mV) and designated as the zeta potential (ξ). Cationic vectors have a zeta potential greater than +10 mV, whereas anionic vectors are less than −10 mV, and neutral vectors are between −10 mV and + 10 mV (Li and Huang, 2008). This charge affects the rate of clearance. The rate of clearance for anionic particles is higher than for neutral or cationic particles, due to phagocytic cells favoring uptake of anionic particles. Cationic particles have increased interactions with cells which results in a decreased circulation half-life compared to neutral or anionic particles (Li and Huang, 2008). This is because of the charge attraction at the negatively charged cell membrane which allows cationic particles to disrupt the lipid bilayers of the cellular membrane and cross the membrane regardless of particle size, shape, or deformability. However, these disruptions in the lipid bilayer periodically create pores that allow for the free passage of proteins, macromolecules, and critical ions, thus disrupting the delicate equilibrium between the interior and exterior of the cell resulting in increased cell toxicity (Leroueil et al., 2008). Interestingly, opsonization, one of the main immune responses against foreign objects, not only affects the hydrodynamic size of a particle, but the charge as well. França et al. showed gold colloids demonstrated observable increases in size and also showed increases in the zeta potential from −38.2 ± 1.2 mV to −16.4 ± 0.6 mV in 30 nm colloids, and − 46.3 ± 0.9 mV to −20.4 ± 1.9 mV in 150 nm colloids (Franca et al., 2011). For this reason, there is an advantage to altering the surface charge on a vector from neutral or weakly negative when administered through intravenous injection to positive when it reaches its destination. The research by Yuan et al. demonstrates this for tumors, wherein zwitterionic particles develop a positive charge in response to environmental stimulus (Yuan et al., 2012), but it is also relevant for sites of HIV-1 induced chronic inflammation (Figure 3). The zeta potential of viral particles is generally negative or weakly negative, e.g., adeno-associated virus (AAV) serotype 2 (AAV2) -9.2 mV (Le et al., 2005). However, it is possible to alter the surface charge of AAV vectors by preparing them with cationic lipids (Fein et al., 2009).

### 2.3 Effect of vector surface coating on biodistribution

The surface coating of the vector determines the hydrophobicity as well as the charge. Ligands and antibodies can be conjugated to the surface of non-viral vectors to actively target certain receptors. While the same can be said of viral vectors (Yao et al., 2017; Thadani et al., 2020), it is more difficult and infrequent. For this reason, surface coatings apply more to non-viral vectors than viral vectors, and this is reflected in the lack of research on modifying viral vector surfaces. Particles with hydrophilic surfaces have a longer circulation half-life and reduced phagocytosis by the MPS, such as polyethylene glycol (PEG) one of the most common surface modifications (Figure 3) (Otsuka et al., 2003). In contrast, particles with hydrophobic surfaces have a significantly reduced circulation half-life and accumulate more in the liver due to greater plasma protein binding. However, PEG-modified particles accumulate primarily in the liver, spleen and bone marrow after a longer time in circulation (Owens and Peppas, 2006). Further, PEGylation interferes with the interactions between particle surface and cell membrane diminishing cellular uptake (Romberg et al., 2008). PEGylation also elicits an immune response and hypersensitivity upon repeated injection, which causes rapid clearance of PEGylated particles by opsonization (Ishida et al., 2006). The numerous problems of PEGylation, i.e., the PEG dilemma, makes the development of alternative surface coatings necessary. Zwitterionic ligands are an example of an alternative material where their hydrophobicity can be altered, which alters their affinity to interact with the cellular membrane and endocytosis kinetics (Yuan et al., 2012).

The surface of a vector is what the immune system will recognize, and repeated administration will most likely result in an immune response against any viral or non-viral vector. Non-viral vectors have an advantage here as it is easier to modify their surfaces to avoid this immune response, whereas even the most uncommon viral vector could potentially have already been observed by a host’s immune system from natural infection, precluding its use.

It is worth noting that the pressure of blood flow can strip the surface coating off a particle, damage the vector or its cargo, and preclude the vector from extravasating toward the target tissue (Hosta-Rigau and Stadler, 2013; Blanco et al., 2015; Cooley et al., 2018; Jarvis et al., 2018; Khor et al., 2018). This may be a key point of consideration when designing vectors given that 35% of people living with HIV on anti-retroviral therapy (ART) experience hypertension (Xu et al., 2017), thus this factor should be taken into account when designing vectors for *in vivo* delivery to PWH.

### 2.4 Effect of vector shape on biodistribution

Shape is a lesser studied factor of vector research, and not relevant for viral vectors given that they are all globular in shape, but generally for non-viral vectors the spherically shaped particles stay in circulation longer. Whereas rod shaped vectors, and other shapes with higher aspect ratios, experience more margination toward the wall of blood vessels more, due to flow-induced rolling (Cooley et al., 2018; Uhl et al., 2018; Da Silva-Candal et al., 2019). Vectors with a larger surface area have increased opportunities to interact with cells lining the walls of the vessel. It is noteworthy, that insufficient binding affinity for the cell membrane of the endothelial cells lining the vasculature, leads to particles being ripped from cell membranes after localization due to architecture-dependent drag forces from blood flow (Khor et al., 2018). Thus, particle extravasation is less likely in patients with hypertension, which occurs in 35% of all PWH on ART (Xu et al., 2017).

### 2.5 Effect of vector deformability on biodistribution

Vector deformability leads to extended circulation half-lives due to the lift forces experiences by these particles from the vascular wall, and their reduced accumulation in the spleen. To demonstrate this, a study showed highly deformably nanoparticles (NPs) accumulated in the spleen at early time points, however these NPs were able to squeeze through interendothelial slits in the spleen and the result was a long circulation lifetime in excess of 30 h (Cui et al., 2014).

### 2.6 Effect of vector degradability on biodistribution

Vector degradability is a determining factor in the distribution of vector cargo. Vectors that degrade in circulation result in stochastic accumulation of cargo within unintended tissues and cells, and subsequently cytotoxicity. This is especially relevant due to the presence of intravascular enzymes, such as nucleases and proteases, which degrade any foreign object administered into the blood stream (Juliano et al., 2009). Furthermore, numerous surface coatings can be applied to prevent NP degradation. Lipid-like coatings, polymeric shells, non-metals or oxide surfaces, layered inorganic metals, as well as polymeric stabilizers/surfactants can be used to this end (Li and Huang, 2010; Kong et al., 2012; Gillich et al., 2013).

### 2.7 Effect of vector cargo on biodistribution

So far, general principles of vector construction have been discussed, but in regards to specifically CRISPR being transported in a vector, the complex structure may require specialized vector design and optimization to ensure efficient encapsulation, protection, and delivery. Further, CRISPR is larger than other gene editing tools, like TALENS or ZFNS, making its use in smaller vectors such as AAV vectors more difficult, which has an upper limit of 4.7 kb, whereas the most commonly used variant of CRISPR, spCas9, is 4.2 kb not leaving enough room for the enzyme, gRNAs, promoters, and other regulatory elements to fit within such as small space. This has prompted investigators to shift to the use of saCas9 (3.1 kb) a much smaller molecule, thereby facilitating a larger payload.

## 3 Vectors

The delivery of CRISPR to the HIV-1 reservoirs is a promising approach to cure HIV. The CRISPR system can be delivered to the reservoirs using vectors such as AAV vectors and nanoparticles. Researchers have published studies that have used these vectors to deliver the CRISPR system to the reservoirs. In this section, we will first discuss the basics of each vector used to deliver CRISPR, the latent HIV-1 viral reservoirs and the barriers that impact delivery, and the most successful vectors to deliver CRISPR to these cellular and anatomical reservoirs.

### 3.1 AAV vectors

An AAV is a small, non-pathogenic virus that can be used as gene therapy vectors. They belong to the family Parvoviridae and are replication-defective, meaning they cannot replicate without the help of a helper virus, adenovirus. AAVs have several features that make them attractive for gene therapy, including the ability to infect both dividing and quiescent cells and the ability to persist in an extrachromosomal state without integrating into the genome of the host cell. AAV vectors have become a popular gene delivery system because of their low toxicity and persistent gene expression and wide range of delivery throughout the body. As such, these have been the most widely used for *in vivo* experimentation which will be discussed throughout section 4 and 5.1. Specifically, adeno-associated virus serotype 9 adeno-associated virus serotype 9 (AAV9) is used a great deal due to its broad tropism, lower immunogenicity compared to other serotypes, and its ability to cross the blood–brain barrier unlike most other serotypes. The variety of AAV serotypes is one of AAV’s greatest strengths, in that each of the serotypes target different tissues, offering delivery options.

However, the disadvantage of using AAV vectors and viral vectors in general is that usually they can only be used once per patient. This is due to the presence of preexisting antibodies that leads to increased distribution of a particular vector to the spleen and significantly decreased distribution to other organs. For example, in monkeys with preexisting antibodies to AAV vectors, a 10-fold increased distribution to the spleen was observed compared to a naïve monkey (Ballon et al., 2020). Although, using a different serotype can overcome this drawback, it limits vector usage. Another drawback of AAV vectors is their packaging capacity, approximately 4.7 kb, which significantly limits the efficiency of how much genetic material you can encapsulate per vector. Some investigators have attempted to compensate for this limitation by separating the payload into two AAV with different serotypes (Dash et al., 2023). Unfortunately, there is no guarantee that the cargo of both vectors will reach the same cells. Although, Dash et al. (2023) showed promising results with the distribution and elimination of HIV-1 within humanized mice using AAV6 and AAV9 containing HIV-1 provirus and CCR5 targeting CRISPR/Cas9. The authors eliminated replication-competent virus in 58% of infected mice.

### 3.2 Other viral vectors

While other viral vectors exist, they are not as widely used for a variety of reasons, such as narrow tissue tropism, higher immunogenicity and toxicity. These include lentiviral vectors, adenoviral vectors, and baculoviral vectors.

#### 3.2.1 Lentiviral vectors

Lentivirus is a type of retrovirus that can integrate its genetic material into the host cell’s DNA, making it a popular choice when genetic integration is desired in gene therapy. Lentiviral vectors are engineered to deliver therapeutic genes to target cells and have been used in clinical trials for HIV-1 treatment. Lentivirus vectors have classically been considered for delivering genetic material, since HIV-1 is a lentivirus, theoretically this type of vector would have the same distribution range, although this can be altered by using alternative envelope genes. Similar to HIV-1 they can also infect dividing and non-dividing cells. Although, their use is limited given that HIV-1 patients may already have antibodies against lentiviral vectors. This is due to lentiviral vectors sometimes utilizing modified HIV-1 genes, such as the envelope and capsid genes. Additionally, integration is usually not a desired trait for treatment, since increasing exposure time to a gene editing tool such as CRISPR increases the chances of off-target effects (Vakulskas and Behlke, 2019). Integrase-deficient lentivirus (IDLV) have been developed to address this concern. In addition to lower risk of insertional mutagenesis and off-target effects, IDLVs are more effective in non-dividing cells since they do not rely on the host cell’s machinery to integrate and replicate, although expression levels are potentially lower than classical lentiviral vectors (Ortinski et al., 2017; Pay et al., 2018).

#### 3.2.2 Adenoviral vectors

Adenoviruses are common respiratory viruses and can be used to make packaging vectors for delivery of genetic information to desired cells. Adenoviral vectors are non-integrating vectors and have commonly been used in clinical trials for cancer treatment. Adenoviral vectors have been used for many years to deliver genetic material and are among the most studied of vectors, since they do not integrate into the host genome. However, as with AAV vectors the immune response limits their use, and if antibodies do not already exist, they will after the host’s first exposure to the vector, and from then on these vectors will elicit inflammation and toxicity. Different serotypes of adenoviral vectors help overcome this barrier somewhat, and also allow for targeting of different cell types and tissues. Despite these drawbacks, adenoviral vectors are relatively easy to mass produce and are widely utilized for vaccines and cancer therapies.

#### 3.2.3 Baculoviral vectors

Baculoviral vectors are derived from a virus that infects insects. They have gained recent prominence as they have similar properties to AAV vectors, such as low toxicity, broad tissue and host tropism, do not integrate genetic information, and can infect both quiescent and proliferating cells. However, their packaging capacity is significantly higher, and can exceed 38 kb. As such they are being used in vaccine development. Also, they have demonstrated transduction into immune privileged tissues, including the brain (Wu et al., 2009). However, as we will discuss more in section 5.2, they must be modified for use in mammals (Figure 1).

### 3.3 Nanoparticles

Nanoparticles are usually in the range of 1 to 100 nanometers and can be made from a variety of materials, including lipids, polymers, and metals. Given the limitations of viral vectors they provide an attractive alternative that can be easily modified to avoid an immune response. Nanoparticles are the most commonly used non-viral vectors for delivering CRISPR due to their high packaging capacity, and low toxicity, however they do suffer from low cargo delivery potential. Nanoparticles can be engineered to deliver therapeutic agents to target cells, and have been used in clinical trials for cancer treatment, gene therapy, and vaccine development. Lipid nanoparticles (LNPs) are a type of nanoparticle that can be composed a variety of lipids and are thus widely utilized in various fields for drug delivery systems due to their biocompatibility and encapsulation potential of therapeutic agents. For example, LNPs have been used in clinical trials for RNA-based therapies, including the coronavirus disease COVID-19 vaccines (Kremsner et al., 2021).

## 4 HIV-1 pathogenesis in susceptible tissues and potential CRISPR delivery methods

HIV-1 infection in humans normally goes undetected for months to years prior to diagnosis. HIV-1 infection of all cell types and tissues occurs within the first few weeks of infection. Given this observation, while ART can control the infection and reverse the immunodeficiency, it is not curative. This results in cellular and tissue reservoirs that contribute to chronic disease. HIV-1 tissue reservoirs can be defined as “a tissue or organ containing cells that contribute to harboring HIV-1 in the setting of ART, regardless of the mechanism maintaining them, i.e., cell quiescence and stability, cell proliferation, low-level viral replication” (Wong and Yukl, 2016). These reservoirs mainly contain exceptionally small numbers of latently infected cells. In the peripheral blood, this has been shown to be approximately one infected cell per one million memory T cells (Chun et al., 1997; Finzi et al., 1997; Chun et al., 1998; Siliciano et al., 2003). These tissue reservoirs are located in virtually all tissues with hot spots having been shown in the GALT, lung, kidney, testes, lymph nodes, and potentially the brain (Estes et al., 2017; Chapon et al., 2021). Within these tissues and in the peripheral blood, the memory CD4 T cell population has been studied as the major cellular reservoir. However, more recently, cells of the myeloid lineage have been shown to contribute as well.

In this section, we discuss the anatomical structure of each of the HIV-1 LVRs and thus the obstacles that vectors must overcome to reach their destination. In so doing, we form a basis to elucidate the mechanics behind successful delivery of vectors to their appropriate destination which will enhance optimization of vector selection, treatment administration, and ultimately treatment efficacy. Vectors that have been shown to target certain tissues with non-CRISPR therapies, but still hold the potential to encapsulate CRISPR are also discussed.

### 4.1 Bone marrow

In theory, the macula occludens loose junctions in the interendothelial clefts of the sinusoidal endothelial cell layer that composes the myeloid bone marrow capillaries would preclude entry of molecules greater than 5 nm from entering the bone marrow interstitium (Figure 3) (Sarin, 2010). However, IV administered vectors larger than the 5 nm still pass this barrier into the bone marrow interstitium, due to the phagocytic nature of the reticuloendothelial cells (Figure 2). This concept allows for imaging of the bone marrow interstitium through bone marrow imaging agents such as systemically administered dextran and polyethylene glycol coated nanoparticles, which are 60 nm in diameter (Figure 2) (Martindale et al., 1980; Illum and Davis, 1987; Daldrup et al., 1999; Daldrup-Link et al., 2000). These agents will accumulate in the myeloid bone marrow interstitial spaces upon transvascular release by reticuloendothelial cells, or spill-over. The permeability of the myeloid bone marrow endothelial cells to blood cell transmigration is reflective of the tissue roles in hematopoiesis and elicitation of an immune response from the MPS. This immune response is contingent on the activation of monocytes and phagocytes of the MPS from their resident interstitial spaces to the site of infection.

### 4.2 Thymic tissues

The contribution of the thymus to the HIV-1 reservoir is somewhat unclear, but the thymus has a clear contribution to disease pathology. Several types of cells have been demonstrated to be infected with HIV-1 and simian immunodeficiency virus (SIV) within the thymus of ART-untreated humans and NHPs, respectively. However, information indicating persistent HIV-1 or SIV infection of the thymus is scarce in ART-suppressed patients.

Delivery to the thymus is difficult as it is protected by the blood-thymus barrier. This barrier is mainly used to block foreign material from contact with cortical T cells, as contact with foreign material causes T cells to undergo apoptosis at this early stage in their development. The blood-thymus barrier is composed of type 1 epithelial reticular cells and their thick basal lamina, continuous endothelial cells joined by tight junctions in the thymic cortex, and macrophages which together preclude permeability of proteins (Bruss and Ely, 2021). Because of these continuous endothelial cells, the size of pores through the blood-thymus barrier is far smaller than required for passage of vectors (Figure 2). Serotypes 8, 9, and 10 of AAV vectors have indicated successful transduction of thymocytes *in situ*. However, transduction efficiency only reached up to 5% of cells (Pouzolles et al., 2020). Likely disruption of the endothelial layer is necessary to achieve efficient delivery of CRISPR to the thymus, and therefore methods demonstrated to disrupt other barriers such as the blood–brain barrier may be beneficial for delivery in the thymus.

### 4.3 Lymph nodes

In ART-treated and untreated patients, lymphoid structures (particularly lymph nodes) house the greatest concentration of lymphoid cells and therefore the greatest concentration of infected cells. Therefore, it is unsurprising that secondary lymphoid organs contain cellular reservoirs during ART in the form of T follicular helper cells (Tfh) within B cell follicles (Pallikkuth et al., 2015; Banga et al., 2016). The B cell follicle is particularly qualified to be a viral reservoir due to the intentional isolation of the compartment from immune surveillance, by cells such as CD8+ T cells, to preserve the highly regulated physiology of the B cell follicle (Connick et al., 2014; Fukazawa et al., 2015). Moreover, inflammation, a hallmark of HIV-1 infection, generates germinal centers and activated Tfh within B cell follicles. Lymph nodes are believed by some to have low drug penetration (Schacker et al., 2000; Fletcher et al., 2014; Rothenberger et al., 2015), however the science is inconclusive and the means by which this would occur remain unclear (Burgunder et al., 2019).

The lymphatic endothelial cell layer is generally considered to be continuous (Farr et al., 1980). Indeed, the junctions between lymphatic endothelial cells consists of materials from adherans and tight junctions, in addition to the presence of a basement membrane (Pfeiffer et al., 2008). However, there is evidence that has come to light to the contrary that calls into question the accuracy of a continuous endothelial cell layer (Figure 2). Electron microscopic images have indicated the presence of 0.1–1.0 μm gaps in the sinus floor of lymph nodes (Clark, 1962; Forkert et al., 1977; van Ewijk et al., 1988; Spalding and Heath, 1989). The leading theory is that these gaps are transient structures because a transmigrating leukocyte is usually contained within the gap walls. Although, these gaps could also be fenestrae that have had their diaphragms torn as well as transendothelial channels. More studies are required to elucidate the structure of the lymphatic endothelial cell layer however these gaps may help explain the increased permeability and retention of 100 nm nanoparticles (Figure 2).

Nanoparticles 100 nm in size have been shown to target the lymph nodes with ideal passivity. Nanoparticles of this size are retained intra-lymphatically, when administered subcutaneously to mice, and then have been shown to disseminate throughout the entire body (Kraft et al., 2017, 2018). The size of the nanoparticles precludes extravasation from lymph vessels, thus inhibiting their discharge into the bloodstream and facilitating nanoparticle transfer between lymph nodes using networked lymphatic vessels (Kraft et al., 2018). Therefore, large nanoparticles are a potential method to deliver therapeutics, such as CRISPR to the lymph nodes to treat HIV-1 infection.

Route of administration may also influence distribution to the lymph nodes. As was demonstrated by Dölen et al. polymeric poly (lactic-co-glycolic) acid (PLGA) NPs subcutaneously or intranodally injected accumulated in local lymph nodes with greater probability than IV injection, which accumulated mainly in the liver and spleen (Figure 2) (Dolen et al., 2020). Dendrimers have shown to accumulate in the lymph nodes of rats. Specifically, the presence of a sialic acid or mannose surface coating increased accumulation in the lymph nodes, with additive effects observed when using both (Figure 2) (Gajbhiye et al., 2013). Nanoparticles with a breviscapine (BVP) surface coating also demonstrated to distribute mainly to the lymph nodes, among several other organs (Liu et al., 2008).

### 4.4 Spleen

The spleen is a controversial viral reservoir as it is a prime candidate for infection due to its role in both the circulatory and immune systems as well as harboring a plethora of diverse HIV-susceptible monocytes, macrophages and CD4+ T cell subsets.

In addition to the liver, the spleen plays a major role in filtering foreign particles through nonspecific uptake. Splenic filtration occurs when particles larger than the width of interendothelial cell slits of the venous sinuses, i.e., 200–250 nm, are presented to the organ. Thus, a safe maximum size limit for nondeformable polymer-decorated spherical particles has shown to be a particle diameter of 150 nm (Figure 3) (Moghimi et al., 2012).

Dendrimers have shown to accumulate in the spleens of rats. Specifically, the presence of a sialic acid or mannose surface coating increased accumulation in the spleen, with additive effects observed when using both (Figure 2) (Gajbhiye et al., 2013). The authors concluded that both sialic acid and mannose are involved in macrophage uptake. Pullulan acetate nanoparticles have also shown high delivery rates and macrophage accumulation in the spleen (Figure 3) (Ravi et al., 2014). Nanoparticles with a BVP surface coating have also demonstrated to distribute mainly to the spleen, among several other organs (Liu et al., 2008). Additional research is needed to elucidate the effectiveness of these systems at delivering CRISPR to the spleen.

It is worth noting that cellular vectors, such as red blood cells (RBCs) have enormous potential especially in delivery to the spleen. RBCs have an innate ability to capture some pathogens and present them to splenic immune cells. This biomimetic delivery method has shown effective delivery of vaccine nanoparticles to the spleen as a result of RBCs natural proclivities (Ukidve et al., 2020). The implications of this for CRISPR delivery are potentially profound, as host-derived RBCs do not elicit an immune response nor do they have a significant carrying capacity limit like viral vectors do. RBCs also have effective delivery to the spleen and a long circulation lifetime, unlike nanoparticles. This is due to the intimate relationship between RBCs and the spleen as well as the self-antigens present on the surface of RBCs.

### 4.5 GI tract

Different parts of the gastrointestinal tract (GIT) contain extensive HIV-1 reservoirs and distinct viral quasispecies (van Marle et al., 2007, 2010; Lerner et al., 2011). A study by Yukl et al. established that the gut harbors 1.2 × 10^9^ infected CD4+ T cells, or 83–95% of all HIV-1-infected cells in the body (Yukl et al., 2010). Thus, the biggest reservoir of HIV-1 in humans is the gut (Estes et al., 2017) The gut-sssociated lymphoid tissues (GALT) specifically houses an abundance of these infected innate immune cells and lymphocytes. Aside CD4 + T cells, macrophages and follicular dendritic cells (FDCs) also contribute considerably to the viral reservoir within the GIT (Brown and Mattapallil, 2014).

No other effective delivery of CRISPR/Cas to the GALT has been reported as of this writing, although, there is potential for several methods to deliver to the GALT. Generally, administration of vectors is thought to be through IV injection. However, this does not need to be the case. Given the wide diversity of tissues types with latent HIV-1, it may be necessary to combine multiple administration routes. For example, oral delivery has been demonstrated for nanoparticles. Microfold cells (M cells) are a prime target to deliver orally administered nanoparticles to the GALT because they function to transport intestinal infectious antigens to the GALT for effective immune response. M cells preferentially take up nanoparticles, specifically those 100-500 nm in diameter. Also, enterocytes take up nanoparticles 20–100 nm in diameter. Thus, particles with a diameter of approximately 100 nm would seem to be preferential to delivery to both M cells and enterocytes (Figure 3) (Cao et al., 2019). It is unclear if these parameters would benefit viral vectors or other forms of treatment administration.

As for active methods of targeting the GALT, nanoparticles with the ligands α4β7 monoclonal antibody (mAb) (Cao et al., 2018) and P2Ns-gambogic acid (GA) (Ganugula et al., 2020) have shown to target these vectors to the GALT lymphocytes. P2Ns-GA target CD71 (transferrin receptor 1), which is present on the Peyer’s patches, intervillous crypts, and intestinal epithelia (Banerjee et al., 1986) in addition to precursor and mature lymphocytes (Ned et al., 2003). Orally administered GA-conjugated nanoparticles dramatically increased delivery of cyclosporine A (CsA) to MRL-lpr mice (an amino acid transporter 1 mutant mice) intestinal lymphoid tissues 4-fold compared to the ligand-free formulation and 18-fold compared to a commercial CsA capsule. For these reasons, GA-conjugated nanoparticles also show potential for delivering the CRISPR/Cas system to the GALT. Whereas α4β7 mAbs target the integrin gut-receptor which have shown selective targeting of a protease inhibitor to gut-homing T cells in primary cells isolated from the macaque ileum. Of course, this delivery vehicle can incorporate the CRISPR/Cas system as well, and mAbs can be conjugated to viral vectors to further increase the amount of available vector (Pearce et al., 2019; Zdechlik et al., 2020).

Additionally, nanoparticles have been shown to efficiently deliver ART to this HIV-1 reservoir (Edagwa et al., 2014). The advantages of polymeric nanoparticles include a higher carrying capacity than viral vectors and malleable surfaces that allow for the addition of ligands that target HIV-1 reservoirs (Amiji et al., 2006; Khalil et al., 2011). Thus, nanoparticles have the potential to improve delivery of ART and other HIV-1 treatments such as CRISPR to the HIV-1 reservoirs (Figure 3). Investigators studied the use of polymer-based pluronic nanocarrier containing an ART drug targeted toward M cells within the GALT (Roy et al., 2015). Similarly, other studies explored the use of polymeric nanoparticles to deliver ART to the GALT reservoir by attaching an M cell-targeting ligand to the surface of nanoparticles (Figure 3) (Ogunwuyi et al., 2016). Further, M cells preferentially absorb nanoparticles (Malinova et al., 2010), however, delivery to the HIV-1-infected cells is very inefficient. The Roy et al. study demonstrated a significant improvement in sustained release of nanoparticle carried ART drug compared to free ART drug, as well as significantly improved ability to inhibit the HIV-1 infection in the GALT compared to the free ART drug *in vitro* (Roy et al., 2015). The Ogunwuyi et al. (2016) study showed that site-specific targeting of ART drug-loaded nanoparticles effectively dispersed and blocked HIV-1 from infecting T cells *in vitro*. These results show promise for delivery of CRISPR to the GALT.

### 4.6 Delivery of CRISPR/Cas9 into tonsils

The tonsils are a poorly studied reservoir for HIV-1 compared to other lymphoid organs. As a result, only CD4+ T cells have been demonstrated to readily support productive infection in this compartment even though they rarely complete the viral replication cycle. This is due to their quiescent nature, but upon stimulation, these cells express virus (Hsiao et al., 2020). Similar to the study of the tonsils as an HIV-1 reservoir, the study of methods to deliver CRISPR/Cas9 to the tonsils is also lacking.

### 4.7 Delivery of CRISPR/Cas9 into the liver

HIV-1 is able to successfully infect and produce virus in liver macrophages (Kupffer cells), hepatocytes, endothelial sinusoidal cells, and stellate cells of the liver (Xiao et al., 2008; Tuyama et al., 2010; Kong et al., 2012). The liver is part of the MPS system and is the largest reticuloendothelial phagocytic system in humans. Thus, it is necessary to tailor a vector to overcome the constraints of this organ. The hepatic venous sinus cortex houses a large number of Kupffer cells, which are the primary retention sites in the liver (Sadauskas et al., 2007). Hepatocytes (Cho et al., 2001) and liver endothelial cells (Chiannilkulchai et al., 1990) potentially have a supporting role.

Hepatic sinusoidal blood capillaries have reticuloendothelial cells that line their capillary walls which have large fenestrae, approximately 100–180 nm in diameter in humans (Figure 2) (Wisse et al., 2008; Sarin, 2010). These fenestrae allow the passage of small lipoproteins from the transvascular flow to the hepatic interstitium (Sarin, 2010). Thus, foreign particles with an extended blood half-life are able to enter the hepatic interstitium through the fenestrae of reticuloendothelial cells or when released by phagocytic reticuloendothelial cells and Kupffer cells (Figure 2). In fact, MPS cells that have phagocytized foreign particles are an enormous reservoir of vectors. The CRISPR/Cas9 system has been successfully delivered to the liver using several methods, including, complexed with gold nanoclusters and further enveloped in a lipid layer; encapsulated within zwitterionic NPs; enveloped within Nanoblades; and with AAV vectors (Figure 3). Nanoblades are murine leukemia virus-like particles (VLPs) encapsulating Cas9-gRNA RNPs. They have shown to be a relevant delivery method as they have shown proficiency at delivering CRISPR components to the mouse liver and potentially human liver (Figure 3) (Mangeot et al., 2019).

Zwitterionic NPs induce a very low immune response and as such are of interest for *in vivo* delivery of therapeutics. The delivery of Cas9 mRNA and gRNA is one such therapeutic of interest. The zwitterionic NP formulation ZA3-EP10 was identified by *in vitro* screening assays for its capacity to deliver gRNA and Cas9 mRNA to cells. *In vivo* results demonstrated continued expression of Cas9 in the liver and kidneys for at least 2 months (Miller et al., 2017). These results demonstrate the potential reliability of the zwitterionic NPs for CRISPR/Cas delivery and therapeutics. Other studies complexed the CRISPR/Cas9 system within gold nanoclusters and further enveloped this in a lipid layer for the purpose of targeting the system to the liver (Figures 2, 3) (Zhang et al., 2019). The gold nanoclusters achieved an *in vitro* editing efficiency of approximately 60% (Zhang et al., 2019).

### 4.8 Delivery of CRISPR/Cas9 into kidney tissue

The kidneys are another major filtration organ where 9% of all exogenous particles end up. Glomerular filtration is the main method of renal excretion and is performed by the glomerular basement membrane (GBM), glomerular endothelial cells, and the slit diaphragms in between podocytes. The podocyte slit diaphragms are the main determinant of size selectivity with a width of approximately 5.5 nm (Schluep et al., 2009), this constitutes the kidney filtration threshold, which particles must exceed to avoid renal excretion. The GBM has pore sizes between 10 and 70 nm, while the endothelial cells have pores larger than 130 nm, thus particles between 70 and 130 nm in size are able to flow from blood past the fenestrae of glomerular endothelial cells but are unable to pass the GBM (Figure 3). Due to these size constraints, particles within this range accumulate in the glomerular mesangium without being released. This accumulation and retention within the glomerular mesangium is designated as an example of the enhanced permeability and retention effect (Scindia et al., 2010).

The role of charge during distribution to the kidneys is not as well understood. While it is clear that a net negative charge exists in the GBM due to the negatively charged glycosaminoglycan (GAG) side chains of the heparan sulfate proteoglycans (HSPGs) that partly constitute the GBM, the knockdown of these species and the reduction of net negative charge of the GBM do not significantly alter filtration. However, cationic and neutral molecular tracers are observed to more efficiently traverse the glomerular filter than anionic tracers. Furthermore, cationic NPs have demonstrated to distribute preferentially to the glomeruli, compared to neutral or anionic NPs, suggesting increased retention (Figure 3) (Elci et al., 2016). Thus, although charge selectivity has demonstrated to be minimally involved in glomerular filtration so far, a greater understanding is needed here to fully elucidate the role of charge selectivity in renal distribution.

### 4.9 Delivery of CRISPR/Cas9 into the lungs

Following IV administration, all vectors migrate to the lungs first, and subsequently to other organs through arterial blood flow. Naturally, particles will be retained by the cells lining the capillary vessel particularly particles greater than 1,000 nm in diameter (Moghimi et al., 2012). To that end, cationic particles commonly distribute to the lungs likely because of electrostatic interactions between blood cells, which form aggregates and are trapped by the small capillaries of the lung (Ishiwata et al., 2000). Using this principle, it is possible to adsorb nanoparticles to the surface of red RBCs to deliver cargo to the lungs, while avoiding elimination by the liver and spleen (Anselmo et al., 2013). Additionally, nanoparticles have been encapsulated in a RBC membrane and their cargo released upon laser activation (Su et al., 2017). While RBCs have not been used for CRISPR delivery, the potential exists and would likely be as effective as previous research using RBCs.

One delivery method that has been explored for lung delivery is lipidoids. Lipidoids have a wide breadth of variation due to a myriad of observed lipid tails. Lipidoids have also been shown to have enormous aptitude for delivering nucleic acid materials (Love et al., 2010; Whitehead et al., 2014; Li et al., 2016; Li and Dong, 2017). Thus, lipidoids have been used to make lipid-like nanoparticles for delivery of nucleic acids to specific anatomical compartments. One such example is the use of lipidoids with O17Se tails housed within nanoparticles that were injected into mice to examine the tropism of these lipidoids by the fluorescence emitted from harvested organs. Lung sections demonstrated high levels of fluorescence indicating a tropism for lung tissue (Wang et al., 2016). The explanation for the observed lung tropism is unclear, but currently under investigation. As such, this method may be useful for CRISPR delivery.

A second delivery method for the lung has been nanoparticles. Different formulations of nanoparticles have predilections for specific organs such as lung tissue (Givens et al., 2018). Furthermore, dendrimers have shown to accumulate in the lungs of rats. Specifically, the presence of a sialic acid or mannose surface coating increased accumulation in the lungs, with additive effects observed when using both. The authors concluded that both sialic acid and mannose are involved in macrophage uptake (Gajbhiye et al., 2013).

### 4.10 Delivery of CRISPR/Cas9 into adipose tissues

Although adipose tissues constitute the largest endocrine organ in the human body, research on the delivery of vectors to adipose tissue has been limited due to the broad distribution of adipose tissue throughout the human body and the significantly reduced population of HIV-1 susceptible immune cells. Furthermore, the upper size limit of the loose junctions in the continuous blood capillaries in this tissue is approximately 5 nm (Sarin, 2010). For this reason, delivery of CRISPR to adipose tissue *in vivo* is very difficult (Figure 3). However, delivery to adipose tissue *in vivo* has been demonstrated with passive and active targeting using peptides conjugated to the surface of plasmids, which show potential for delivery of non-viral plasmids (Figure 3) (Shen et al., 2018; Chung et al., 2019).

The route of administration has a prominent effect on distribution of vectors to the adipose tissue.” This makes the sentence more concise. Local delivery to adipose tissue, via intra-white adipose tissue (WAT) or intra-brown adipose tissue (BAT) injection, has shown to greatly increase the accumulation in the WAT and BAT, respectively. Delivery of genetic materials to adipose and the benefit of each route of administration for adipose is reviewed further by Romanelli and Mac Dougald (2020).

### 4.11 Delivery of CRISPR/Cas9 into peripheral blood

Resting T cells in circulation are a well understood reservoir for HIV-1 during suppressive ART (Lee and Lichterfeld, 2016). The central (Tcm) and transitional memory (Ttm) CD4+ T cell subsets make up the biggest fraction of latently infected CD4+ T cells in the peripheral blood of ART-suppressed individuals (Chomont et al., 2009; Yukl et al., 2013). The convenient accessibility of peripheral blood makes delivery of CRISPR/Cas the simplest of any reservoir. Thus, a large variety of delivery methods are available to transduce CRISPR/Cas into *in vivo* or *ex vivo* circulating T lymphocytes (Figure 3). These include physical methods such as electroporation and microinjection of plasmid DNA, mRNA, or RNP (Schumann et al., 2015; Gwiazda et al., 2016; Su et al., 2016; Seki and Rutz, 2018; Hultquist et al., 2019), viral vectors such as lentiviral vectors (Kaminski et al., 2016; Bella et al., 2018), retroviral vectors (Huang et al., 2019), adenoviral vectors (Li et al., 2015), AAV vectors (Kaminski et al., 2016), and chemical methods such as lipid-based nanoparticles (Figure 3) (Lokugamage et al., 2019). Extracellular vesicles have also been shown to efficiently deliver the CRISPR/Cas system against integrated HIV-1 provirus in the model cell line HEK293FT cells and cause viral disruption (Campbell et al., 2019). Therefore, they show potential for CRISPR/Cas delivery to lymphocytes and viral disruption.

### 4.12 Reproductive tract

The barriers a vector experiences for reproductive tract delivery are different depending on sex. The blood-testes barrier (BTB) exists for males, while the blood-follicular barrier (BFB) exists for females.

#### 4.12.1 Blood testes barrier

For males, the BTB is composed of tight junctions, gap junctions, and adherens junctions between sertoli cells of the seminiferous tubule, located just above the basement membrane (Vogl et al., 2008). The blood capillary type for the testis is fenestrated with diaphragmed fenestrae capillary type and a maximum pore diameter of 6–12 nm (Figure 3) (Sarin, 2010).

More research is needed to fully understand the complexities of the size constraints of the BTB. If 12 nm is in fact the upper limit for delivery to the testes in a non-diseased state as well as during acute and chronic inflammation, then methods must be developed to improve testes delivery by temporarily enlarging pores in the BTB to mediate vector recipience. The preliminary research for this idea is already underway. Scrotal heat stress and pulsed unfocused ultrasound (Li et al., 2020) as well as testes injected bacterial magnetic particles (Wang et al., 2017) have already indicated that these methods increase recipience to the testes providing great promise for the delivery of CRISPR to the testes as well (Figure 2).

#### 4.12.2 Blood follicle barrier

Delivery from the blood stream into the interior of the ovarian follicle is restricted by the BFB. The BFB is constituted by the membrana granulosa, follicular basement membrane, thecal interstitium, sub-endothelial basement membrane, and vascular endothelium (Zhou et al., 2007). While different layers of the BFB permit entry to different sizes of molecules, overall the BFB is permeable to molecules with a molecular weight less than 500 kDa (Figure 2) (Cran et al., 1976). Additionally, the BFB has shown to be more permeable to positively charged and smaller molecules without ovulatory stimulus, but larger and negatively charged molecules are still able to enter upon ovulatory stimulus in mouse ovaries (Figure 2) (Hess et al., 1998).

Interestingly, for females, the blood capillary type differs based on the developmental stage of the ovarian follicle. During folliculogenesis, fenestrated blood capillaries are present with the maximum pore diameter of about 12 nm for pre-ovulatory follicles (Figure 3) (Sarin, 2010). Whereas, developed ovarian follicles have a continuous blood capillary type with loose junctions present and an maximum pore diameter of about 5 nm (Sarin, 2010). This discrepancy in permeability is likely due to the mercurial structure of the follicular basement membrane during folliculogenesis (Rodgers et al., 2003; Siu and Cheng, 2012).

Overall, there is a lack of research analyzing delivery of the CRISPR/Cas system to the female reproductive tract.

### 4.13 Delivery of CRISPR/Cas9 to the brain

Microglial cells are the primary immune cells of the brain, and also the main viral reservoir within the brain. Understandably so, as they represent a highly sustainable reservoir due to their year’s long half-life (Reu et al., 2017), ability to mitotically divide (Lawson et al., 1992), and potentially high susceptibility to HIV-1 infection (Cenker et al., 2017). The main challenge for elimination of HIV-1 latent reservoirs in the central nervous system (CNS) with CRISPR/Cas9 is the delivery of the system across the blood–brain barrier (BBB). The BBB is mainly comprised of endothelial cells, but also consists of astrocytes, adjacent neurons, microglial cells, and pericytes (Chen and Liu, 2012). The polarized endothelial cells of the BBB facilitate the influx of molecules across the BBB through passive diffusion and active transport mechanisms. However, these endothelial cells are highly selective due to the presence of tight junctions and adherent junctions. Small intercellular pores in tight junctions make passive diffusion a possibility for molecules that are hydrophobic in nature with a 500 Da or less molecular weight, but few vectors meet these criteria.

The hydrophobic nature of the BBB makes it more permeable to lipophilic molecules, whereas it is generally impermeable to hydrophilic molecules, unless the appropriate channels are expressed in the endothelial cells and astrocytes that constitute the BBB (Figure 2). Thus, very small hydrophobic molecules may passively diffuse across the BBB, but vectors and drugs are subject to expulsion from the brain parenchyma by membrane transporters which include the P-glycoprotein-type multidrug resistance efflux pump and multi-specific organic anion transporter. Even hydrophobic molecules experience an additional barrier to entry into the brain with the presence of inter and extra-cellular hydrophobic enzymes in the endothelial cells (El-Bacha and Minn, 1999).

Despite the lack of fenestrations in endothelial cells, vectors still manage to penetrate into the brain. The latest theory is that these vectors pass through poorly developed or damaged areas or using dendrites and neuronal axons to penetrate the BBB (Oberdorster et al., 2009; Lankveld et al., 2010). Damage to the BBB by HIV-1 infection has been studied more so than other tissue barriers given the importance of the CNS as a reservoir. The general effect of this damage, as with other endothelial dysfunctions, is that the barrier is permeabilized. More research is needed to understand to what extent this permeability occurs, but the effects are likely to vary depending on the extent of disease severity. Interestingly, drugs of abuse, a common problem among those infected with HIV-1, exacerbate disease progression and damage to the body (Shirazi et al., 2013; Parikh et al., 2014).

To overcome the obstacles to delivery that the BBB presents, shrinking endothelial cells, thus opening up endothelial tight junctions has been demonstrated to allow paracellular transport of particles less than 20 nm across the BBB (Figure 2) (Azad et al., 2015). Other means of disrupting the BBB include the use of hyper-osmotic mannitol to facilitate particle penetration across the BBB (Kroll et al., 1998).

General aspects of vectors that have been found to be beneficial to crossing the BBB include altering the size and shape of the vector, as well as attaching to ligands of cell receptors to facilitate active transport. Small particles (200 nm or less), deformable particles, and rod shaped particles have shown to be beneficial to passively diffuse through the BBB (Figure 2) (Nowak et al., 2020). Some drugs, such as L-DOPA, have demonstrated to use the L-type amino acid transporter 1 (LAT1) receptor in the BBB. The surface coating BVP (Liu et al., 2008) and apolipoprotein E (ApoE) (Dong et al., 2014; Bertrand et al., 2017; Dal Magro et al., 2017; Chen et al., 2019) can also be delivered to the brain (Figure 3). Vectors with a surface containing the ApoE ligand target the low-density lipoprotein receptors, which leads to delivery across the BBB in some instances.

While the above research has shown in theory how vectors should be designed to facilitate effective entry into the CNS, in practice it is more difficult to account for the variability seen *in vivo* across vectors, patients, and disease states. Thus, an explanation for the effective entry of some vectors requires more research. Nanoparticles have the potential to cross the BBB and deliver CRISPR to the CNS. The small size and lipophilic properties of lipid nanoparticles makes them endocytose into the CNS with efficiency (Figure 3).

## 5 Successful cases of CRISPR delivery to the HIV LVRs

### 5.1 AAV vectors

A series of studies by K. Khalili and colleagues has shown the use of AAV vectors to deliver anti-HIV-1 CRISPR therapeutics into a number of animal models and more recently as a first in human studies. In a humanized mouse model, AAV9-CRISPR-SaCas9 used in combination with long-acting slow-effective release (LASER) ART has been reported to eliminate the latent HIV-1 reservoir from the bone marrow, thymus, lymph nodes, spleen, GIT, tonsils, peripheral blood, brain, lungs, kidneys, liver, and male reproductive tract of 2/7 humanized mice. No viral rebound was observed after treatment interruption in the mice using ultrasensitive HIV-1 nucleic acid detection methods and a lack of viral transmission from infected, dual-treated mice to uninfected untreated mice further confirming successful viral elimination (Figure 1) (Dash et al., 2019). This group went on to use AAV vectors to deliver CRISPR/SaCas9 to many LVRs, of an SIV-infected rhesus macaque model in a proof of concept study that showed CRISPR could be delivered to the same anatomical reservoirs as in humanized mice and prevent rebound of SIV 1 week after *in vivo* CRISPR treatment (Figure 1) (Mancuso et al., 2020). More recently, this research has been pursued by the biopharmaceutical company Excision BioTherapeutics in collaboration with Khalili and colleagues. This collaboration performed a preclinical safety, biodistribution, and efficacy study in an SIV model using the treatment EBT-001, a SaCas9 endonuclease with SIV long terminal repeat (LTR) and Gag targeting gRNAs encapsulated within an AAV9 vector, the same treatment used by Mancuso et al. (Figure 1). They demonstrated that just one IV injection of EBT-001 penetrated the spleen, mesenteric lymph nodes, colon, bone marrow compartment, and blood as shown by quantitative polymerase chain reaction (qPCR). Polymerase chain reaction (PCR)-based exclusion assays observed breakages in SIV DNA throughout most tissues in samples collected at 3 and 6 months post treatment administration (Figure 1) (Burdo et al., 2023). This successful preclinical trial has led to an ongoing phase I/II clinical trial using the HIV therapeutic candidate, EBT-101, which uses two similar gRNAs as in the SIV model, targeted against the HIV LTRs and Gag and uses the saCas9 and AAV9 vector for packaging as before. Forty eight weeks into this clinical trial no participants experienced any serious adverse events or dose-limiting toxicities during the trial. Additionally, EBT-101 was detected in the blood of all participants (Presti et al., 2023). Indeed, AAV vectors are very prominent in the delivery of CRISPR, including HIV research (Figure 1).

A number of other investigators have shown other important end points of AAV vectors used in the delivery of CRISPR therapeutics. One study showed that AAV vectors were able to efficiently deliver the CRISPR/Cas system into mice to disrupt the HIV-1 provirus. Tg26 and Eco-HIV mice intravenously injected with quadruplex gRNAs/SaCas9 AAV-DJ/8 targeted against the LTRs, Gag and Pol regions showed cleavage of HIV-1 proviral DNA, as determined by PCR genotyping of the liver, lungs, brain, spleen and colon (Figure 1). Notable reduction of virus replication was also observed by live bioluminescence imaging (Yin et al., 2017). Another advantage of AAV vectors is that it can be directed to preferentially deliver cargo to specific cell types, such as astrocytes. Kunze et al. (2018) demonstrated that the transduction efficiency of synthetic AAV vector carrying CRISPR/Cas9 was higher in terminally differentiated human astrocytes compared to neurons using primary human brain cells and human organoids (Figure 1).

### 5.2 Other viral vectors

Due to their limitations, Adenovirus use has primarily been restricted to the peripheral blood, although CRISPR/Cas9 has been shown to be delivered to the liver of mice as well (Wang et al., 2015). This successful delivery to mouse liver occurred in spite of an immune response to adenovirus as well as the spCas9 machinery in the liver. Successful editing of the target gene was observed but liver damage did occur as a result of this treatment.

Lentivirus vectors are primarily used *in vitro*, whereas *in vivo* it is mostly used in peripheral blood (Kaminski et al., 2016; Bella et al., 2018) (Figure 1). This is due to safety concerns, since lentiviruses integrate foreign DNA into the chromosomes of host cells thus potentially causing unwanted insertion mutations, oncogenicity, and genotoxicity. Lentiviral vectors can be further reviewed by Dong and Kantor (2021).

Baculoviral vectors are inactivated by the immune system in mammals. This limits their use for systemic applications. However, they can be used in immune-privileged tissues or locally with reduced exposure to the immune system. A study by Zhu et al. (2019) developed a hybrid nanoparticle-baculoviral vector that used serum inactivation as an “off” switch and magnetic fields as an “on” switch for tissue-specific gene editing. This was used *in vivo* to encode CRISPR/Cas9 targeted against the Vegfr2 gene in the brain causing reduction of the targeted gene (Figure 2). This method combines the advantages of nanoparticle delivery with the high gene-carrying capacity of baculoviruses, offering a promising tool for precise *in vivo* genome editing (Figure 1).

### 5.3 Nanoparticles

Research using nanoparticles have been used to deliver CRISPR to the bone marrow, lymph nodes, spleen, GIT, peripheral blood, and brain. While pullulan acetate nanoparticles show high delivery rates and macrophage accumulation in the spleen, liver, and lymph nodes (Figure 2) (Ravi et al., 2014). Magnetic nanoparticles have been shown to deliver CRISPR across the BBB by application of an external magnetic field *in vitro* and *in vivo*. On-demand controlled release of CRISPR/Cas9 targeted against the HIV-1 LTR was developed for delivery across the BBB *in vitro* (Figure 1) (Kaushik et al., 2019). Stimulated expression of CRISPR/Cas9 upon application of magnetic field stimulation showed reduction of HIV-1 LTR expression levels in latently infected microglial cells (Figure 1). Gold nanoparticles are conducive for drug delivery to the CNS because of their small size, malleable shape, and the ease of modifying ligands (Figure 3). *In vivo* delivery and successful editing of the mGluR5 gene with CRISPR/Cas9 and Cas12a has been demonstrated in local regions of the brain of a fragile X syndrome mouse model with CRISPR-Gold (Lee et al., 2018). Avenues of administration can also impact delivery and efficacy of a treatment to the brain. One such study by Niu et al. involved poly (β-amino ester) nanoparticles in a gel material administered to pig vaginas (Figure 3). The authors found that targeting the CRISPR/Cas system against porcine endogenous retroviruses (PERVs) resulted in a significant reduction in the PERV copy number within the vaginal epithelium. Furthermore, the topically delivered Cas proteins were expressed only in the vagina/cervix and did not migrate to adjacent organs. (Figure 1) (Niu et al., 2020). The length of time a vector is present in the body can also impact the efficacy of the treatment, but this is not always something that is measured. One study using zwitterionic NPs to deliver of CRISPR/Cas9 to the liver and kidneys *in vivo* demonstrated continued expression of Cas9 in the liver and kidneys for at least 2 months after delivery of CRISPR (Miller et al., 2017). For example, LNPs have been used in clinical trials for RNA-based therapies, including the COVID-19 vaccines. Investigators also recently engineered modified LNPs to efficiently deliver Cas9/sgRNA RNP complexes into cells within the brain, muscle, liver, and lungs (Wei et al., 2020). As such, they may also be useful for HIV-1 therapeutics (Figure 3).

## 6 Discussion

The future outlook to target the HIV-1 LVRs with CRISPR machinery has great potential. While the current delivery methods, such as viral vectors and nanoparticles, have respectable efficiency when targeting the HIV-1 LVRs, they still have significant draw backs such as immune response against viral vectors and highly variable tissue penetration among nanoparticles. So, the latest developments in targeted delivery, i.e., cell-based targeting systems such as erythrocytes, macrophages, and even T cells and dendritic cells may some day soon become the dominant methods for delivery of CRISPR due to their biocompatibility and tissue penetration. T cells and dendritic cells were not covered in this review due to a lack of research stemming from the complexity of their isolation and manipulation, shorter lifespan, and potential for autoimmunity (Kamath et al., 2002; Agosto et al., 2015; Mason et al., 2015; Audiger et al., 2017; Baliu-Pique et al., 2018; Fu and Jiang, 2018). More research is needed to elucidate the effectiveness of these cutting-edge methods involving delivering CRISPR to LVRs, as well as the permanent disabling of the integrated HIV-1 provirus. However, similar to CRISPR itself, it is more than likely that the next major development in making delivery of gene editing tools clinically safe will quickly gain popularity when it is first demonstrated for use with CRISPR. The next priorities for safely developing *in vivo* CRISPR delivery methods is further elucidating the architecture of each tissue type at each stage of HIV-1 infection and how this architecture alters the permeability and retention of each type of vector. Thus, understanding the mechanisms behind cell and tissue transduction are of paramount importance to developing effective and clinically safe gene editing as they will lay the foundation for how all vectors will be designed for delivery in patients.

Since their development, ART drugs have been the primary treatment for HIV-1 infection. ART has transformed one of the most deadly viruses in human history into a chronic disease and has saved the lives of tens of millions of people. However, the establishment of the LVR soon after infection necessitates a lifetime commitment to ART consumption. The inaccessibility of ART drugs for some, and the difficulty of ART adherence make eliminating the HIV-1 LVR essential. The CRISPR/Cas system is, by far, the most simple and efficient gene editing method available, thus it carries great promise in its application to HIV-1 therapy. However, the large size, ~4.1 kb, of the most commonly used endonuclease SpCas9 inhibits delivery efficiency. The smaller SaCas9, ~3.1 kb, moderately alleviates this impediment to delivery. The efficient delivery and specific targeting of the CRISPR/Cas system are necessary for the successful translation of this research into a clinical setting. Failure to specifically target the DNA of interest will result in off-target effects and cell toxicity (Link et al., 2018; Sullivan et al., 2020; Atkins et al., 2021). The probability of off-target effects increases with Cas exposure time, therefore, transient expression vectors such as IDLVs, AAV vectors, or chemical methods are preferable to permanent expression vectors, e.g., wild-type lentiviral vectors, to mitigate off-targets effects (Wu et al., 2014; Choi et al., 2016). The successful translation of CRISPR into a clinical setting is dependent on the improvements to vehicles to deliver CRISPR/Cas safely and effectively. Also, combining the CRISPR/Cas system with new strategies for delivery and elimination of HIV-1 has made CRISPR/Cas overcome its restrictions, as was demonstrated when CRISPR/Cas used with LASER-ART showed remarkable elimination of HIV-1 from viral reservoirs (Figure 1) (Dash et al., 2019). Importantly, the investigators performing these investigations demonstrated reduction of HIV in every primary HIV tissue reservoir, which will be necessary to prevent HIV resurgence and viral escape, otherwise retreatment becomes inevitable. As all tissue and cell types have different uptake parameters, the specifics of which are largely unknown, and every viral reservoir must receive a therapy for permanent effect, further research is required for the most effective delivery of HIV cure strategies to viral reservoirs. Further developments such as these will improve the delivery and targeting specificity of the CRISPR/Cas system. Overall, although significant limitations to the CRISPR/Cas system exist that necessitate their improvement before clinical translation of the technology, CRISPR shows enormous promise as a potential treatment and cure to HIV-1 infection.

## Author contributions

TG: Conceptualization, Visualization, Writing – original draft, Writing – review & editing. SE: Writing – review & editing. IS: Writing – review & editing. WD: Writing – review & editing. MN: Conceptualization, Project administration, Supervision, Visualization, Writing – review & editing. BW: Conceptualization, Funding acquisition, Project administration, Supervision, Writing – review & editing.
